# Editorial: Linkage between membrane composition, cellular functions and membrane-mediated stress

**DOI:** 10.3389/fgene.2023.1216425

**Published:** 2023-05-18

**Authors:** Pradipta Saha, Mohan Chandra Joshi, Sangeeta Tiwari, Rahul Saxena

**Affiliations:** ^1^ Department of Microbiology, The University of Burdwan, Burdwan, India; ^2^ Multidisciplinary Centre for Advanced Research and Studies, Jamia Millia Islamia University, New Delhi, India; ^3^ Department of Biological Sciences and Border Biomedical Research Centre, University of Texas at El Paso, El Paso, TX, United States; ^4^ Department of Biochemistry and Molecular and Cellular Biology, Georgetown University Medical Center, Washington, DC, United States

**Keywords:** membrane-protein association, lipid rafts, detergent-resistant membrane, *Agrobacterium tumefaciens*, *Lactobacillus acidophilus*, Tafazzin

Biological membranes are dynamic and complex structures. These are vital for maintaining the functions and integrity of cells in diverse forms of life. The proteome (transmembrane and peripheral membrane proteins) and lipidome (various lipid species, including zwitterionic and anionic phospholipids) forming biological membranes determine their functional characteristics. Besides, the peptidoglycan layer provides rigidity to the biological membrane. There are compelling scientific investigations that advocate the assembly of phospholipids and proteins complexes in membranes as distinct micro-domain structures, referred to as “lipid rafts”. These lipid rafts in the membranes are resistant to anionic detergent treatment and referred to as “detergent resistant membrane (DRM)”. The composition and function of lipid rafts is well investigated in eukaryotic cell membranes, and eukaryotic lipid rafts contain phospholipids, sphingolipids and cholesterol. Though lipid rafts are less explored in prokaryotes, it appears that the organization of bacterial membranes follow similar principle. However, the compositions of lipid raft in bacteria differ considerably from their eukaryotic counterparts. A restricted knowledge about lipid rafts is obtained from bacteria such as *Bacillus subtilis*, *Staphylococcus aureus* among Gram positives and *Escherichia coli*, *Borrelia burgdorferi* and *Pantoea sp*. YR343 among Gram-negative bacteria.

In addition, the association between acidic phospholipid, cardiolipin (CL), and membrane proteins plays a crucial role in regulating various critical cellular functions. In bacteria, CL controls the activity of membrane-associated proteins involved in sporulation, biofilm formation, and transport across the cellular membrane, and bacterial pathogenesis. In eukaryotes, CL maintains the architecture of mitochondrial membranes by regulating the activity of enzymes and proteins involved in critical functions, including acidic phospholipid-induced apoptosis, oxidative phosphorylation, and regulation of respiratory complexes. The study of lipid-protein complexes links distinct crucial cellular processes, and in fact, any abnormal changes in the membrane composition can cause cellular stress, eventually leading to cell death.

Czolkoss et al. explored the proteome profile of DRM from a Gram-negative plant pathogenic bacterium *Agrobacterium tumefaciens* and provided evidence for the functional compartmentalization of its membrane. To achieve this, the authors standardized a detergent cocktail-based membrane solubilization method, followed by low-speed centrifugation to achieve phase separation between DRM and detergent-sensitive membrane (DSM). In the former fraction, three putative SPHF (named after the proteins Stomatin, Prohibitin, Flotillin, and HflK/C) homologs, hflC, hflK, and atu3772, were identified. The SPHF protein family members are considered markers for lipid raft and are found evolutionary conserved in bacteria, archaea, and eukaryotes. The homolog proteins expressed as recombinant FLAG-tagged variants and their cellular localization were confirmed. In addition, the authors isolated membrane-associated proteome of virulence-induced and non-induced cultures, separated into DRM and DSM, and assessed them by LC-MS/MS approach. Specifically, the DRM fraction contains virulence-related proteins, such as type IV and type VI two-component secretion systems. The spatial localization of these two-component systems was confirmed using immunoblotting and immunofluorescence-based techniques. The results presented in the manuscript suggested the existence of distinct lateral segregated micro-domains of membranes that harbors specific physicochemical parameters to support unique biochemical processes (in this case, infection in plants by pathogenic bacteria).

Jeon et al. successfully attempted to improve the thermal adaptability of a wild-type *Lactobacillus acidophilus*, EG004 strain (through the adaptive laboratory evolution method) with minimal changes in the genetic background. *L. acidophilus* probiotic strains have a huge demand due to their beneficial effects on various human diseases, such as innate immunity, inflammatory bowel disease, and colon cancer. However, the low thermal stability in bacteria limits its use in the industrial sector (particularly in making animal feed additives). The authors engineered an *L. acidophilus*, EG008 strain, which retained all the beneficial properties of wild-type bacteria, but with a notable feature of improved thermal resistance (from 65°C to 75°C). The authors provided the whole genome sequencing and comparative genome analysis data, which confirmed that the newly developed strain is genetically identical to wild-type bacteria with only two single nucleotide polymorphisms (SNPs) variations. One non-synonymous SNP change corresponds to the substitution of amino acid residue serine by threonine, residing at 435 positions in UDP-N-acetyl muramyl-L-alanine-D-glutamate ligase (product of murD gene) enzyme. The second SNP variation was present in the non-coding region flanked by two genes; one synthesizing galactose-1-phosphate uridylyltransferase and another L, D-transpeptidase. The UDP-N-acetyl muramyl-L-alanine-D-glutamate ligase and L, D-transpeptidase enzymes are involved in the peptidoglycan layer synthesis, which is associated with the rigidity of the cell wall. The authors speculated that the combined effect caused due to 1) enzymatic stability of UDP-N-acetyl muramyl-L-alanine-D-glutamate ligase conferred by an incorporated change in amino acid residue and 2) manipulating the gene expression level of L, D-transpeptidase enzymes, makes cell wall of newly-developed *L. acidophilus*, EG008 strain relatively more rigid, thus allowing increased thermal tolerance in bacteria.

Jagirdar et al. investigated the ability of an acyltransferase, tafazzin (TAZ), in particular a human variant lacking exon 5 (∆5 isoform), to restore cardiolipin composition, rate of cellular proliferation and differential gene expression in TAZ-deficient C6 Glioma cells (a cell line isolated from the brain of a rat with Glioma). The full-length human tafazzin gene contains 11 exons, which could express at least four variant transcripts. Tafazzin is involved in cardiolipin remodeling, and it does so by mediating the exchange of fatty acids between phospholipids and mono-lyso-phospholipids produced during oxidative stress in the cells. Additionally, tafazzin plays a role in cell proliferation and regulates gene expression profiles. The data presented in the manuscript revealed that a lack of tafazzin in C6 Glioma cells leads to substantial changes in the expression profile of genes related to lipid metabolism. It includes the dysregulation of genes that participate in the cholesterol biosynthesis pathway. The results presented here confirmed that re-expression of full-length rat tafazzin restored cardiolipin composition, cell proliferation rate, and gene expression profile similar to the C6 Glioma cells carrying intact levels of cellular tafazzin protein. Like full-length rat tafazzin, expression of the human ∆5-isoform restored cardiolipin composition, but it showed weak activity in restoring the rate of cell proliferation and reverses the gene expression profile only to a lesser extent. The authors suggested that differential gene expression in TAZ-deficient C6 Glioma cells expressing the human ∆5-isoform might have been linked with epigenetic regulation.

The biological membrane acts as a junction where lipids and proteins interact. A significant but relatively unaddressed question is the identification of how lipid species (comprising lipidome) are involved in organizing a wide variety of proteins (comprising proteome) to form molecular machines involved in crucial cellular processes. The research article by Czolkoss et al., cited in the current editorial, identified how virulence traits in *A. tumefaciens* compartmentalized into specific microdomain-like structures present in the membrane. Another unknown is how any alterations in membrane components could influence cellular functions. Jeon et al. presented an example of how changing the rigidity of cell membranes allows thermal tolerance in *L. acidophilus* bacteria. Jagirdar et al. correlated the composition of lipid content (particularly an anionic phospholipid, cardiolipin) with differential gene expression in C6 Glioma cells deficient in tafazzin, an acetyltransferase involved in cardiolipin remodeling. The studies could be crucial to understanding the impact membranes have on critical cellular events.

